# Evidence of the Practice of Self-Medication with Antibiotics among the Lay Public in Low- and Middle-Income Countries: A Scoping Review

**DOI:** 10.3390/antibiotics9090597

**Published:** 2020-09-12

**Authors:** Adeel Aslam, Márió Gajdács, Che Suraya Zin, Norny Syafinaz Ab Rahman, Syed Imran Ahmed, Muhammad Zeeshan Zafar, Shazia Jamshed

**Affiliations:** 1 Department of Pharmacy Practice, Kulliyyah of Pharmacy, International Islamic University Malaysia, Kuantan 25200, Malaysia; adeel.aslam224@gmail.com (A.A.); chesuraya@iium.edu.my (C.S.Z.); norny@iium.edu.my (N.S.A.R.); 2 Department of Pharmacodynamics and Biopharmacy, Faculty of Pharmacy, University of Szeged, 6720 Szeged, Hungary; gajdacs.mario@pharm.u-szeged.hu; 3 Institute of Medical Microbiology, Faculty of Medicine, Semmelweis University, 1089 Budapest, Hungary; 4 Department of Pharmacy Practice, School of Pharmacy, International Medical University, Bukit Jalil, Kuala Lumpur 57000, Malaysia; 5 College of Pharmacy, University of Sargodha, Sargodha 40100, Pakistan; shanmughal11@gmail.com; 6 Qualitative Research-Methodological Application in Health Sciences Research Group, Kulliyyah of Pharmacy, International Islamic University Malaysia, Kuantan 25200, Malaysia

**Keywords:** self-medication with antibiotics, SMA, scoping, review, low- and middle-income countries

## Abstract

The current scoping review is an attempt to explore the key reasons, determinants, patterns and prevalence related to self-medication with antibiotics (SMA) among the lay public. An online search was conducted using Google Scholar, Science Direct, ProQuest and PubMed. A two-phase mapping approach was used. In the first phase, studies were screened. In the second phase, the data were extracted from selected studies followed by the assessment of data quality. A total of 24 studies were included; 20 were cross-sectional, 3 were qualitative and one was observational. The most common indications were flu, cough, common colds, sore throat, diarrhea, toothache and fever. The most common determinants reported were past good experience and suggestions from friends or relatives. The use of SMA was observed to be more frequent in younger aged individuals belonging to low- or middle-income groups. The prevalence rate was reported to be high among the South Asian lay public and may be a major contributor to antibiotic resistance. In conclusion, this scoping review identifies a need for education campaigns and mass media campaigns to strengthen lay public awareness about the side effects and risks associated with SMA. In addition to this, there is a need to implement strict policies by government agencies to restrict over the counter availability of antibiotics.

## 1. Introduction

The increasing level of antimicrobial resistance (AMR) is a significant trend seen throughout the globe, and this phenomenon may affect general public health at all levels of healthcare [1]. It decreases the potency of antibiotics during the treatment of infectious ailments and extends the duration of illness. Also, extensive use of these drugs increases the emergence and spread of drug-resistant microorganisms among patients [2]. Over the last few decades, the treatment of infections with antibiotics as primary care among the population has changed dramatically. In addition, some individuals use antibiotics more frequently which results in more multidrug-resistant infections. An obvious relationship has been shown between the amount of antibiotics utilized and the prevalence of bacterial resistance [3]. When it comes to the clinical significance and overall mortality associated with drug-resistant bacterial pathogens, “ESKAPE” bacteria (E: *Enterococcus faecium*, S: *Staphylococcus aureus* and *Stenotrophomonas maltophilia*, K: *Klebsiella pneumoniae* or recently C: *Clostridioides difficile*, A: *Acinetobacter baumannii*, P: *Pseudomonas aeruginosa*, E: *Enterobacter* spp. and *Enterobacteriaceae*) should be highlighted as the most important concerns [4,5]. These multidrug-resistant organisms are the most common cause of healthcare-associated infection in low and middle-income countries (LMICs), and these multidrug-resistant organisms increase the morbidity and mortality rates [6]. A study performed in LMIC (Syria) showed how antibiotic-resistant nosocomial infections are increasing the number of deaths, and this resistant nosocomial infection is also spreading in European countries through refugees, which could turn into a global threat [7]. Based on this evidence from LMICs, it appears that the lay public are using antibiotics for Self-medication (SM) and this practice is leading to drug-resistant infections or organisms. Self-medication (SM) can be described as the utilization of drugs to treat ASL-diagnosed disease or symptom or long-term use of prescribed medicine for a chronic or recurrent disorder [8]. People may self-medicate with antibiotics primarily for oral infection bronchitis, congestion, influenza and urinary discomfort [9]. Most people who undergo SM with antibiotics obtain then from community pharmacies (without any medical prescriptions), use leftover antibiotics from a previous prescription or obtain them from relatives, friends or other people who have obtained them from the Internet or online [10].

However, the WHO (World Health Organization) has confirmed that SMA is an unhealthy behavior and an inappropriate method of utilizing these medicines. Furthermore, according to WHO reports, most people who used antibiotics for themselves do not have the appropriate knowledge about dosage and use them for an improper duration of time. This practice may lead to the development of drug resistance [2,11]. These practices defined by WHO most commonly involve LMICs, and the prevalence rate for self-medication with antibiotics (SMA) is also high among the lay-public [12,13,14]. Therefore, there is an obvious need for the synthesis of available literature in the form of a well-designed scoping review study on the utilization of antibiotics by the lay public (a person or group of people who do not have expert knowledge on a particular subject) [15] in LMICs to aid in mapping evidence, identify research gaps and guide future research. Scoping reviews are a relatively new type of research review, providing a tool for summarizing literature in a specific topic area such as health system quality reporting [16]. Scoping reviews are, to some extent, linked to systematic reviews, due to the fact that both are accustomed to methodically organizing and explain a body of literature. Systematic reviews make an effort to answer a well-defined question, and they frequently use specific methodologies to evaluate the standard of the included content. On the other hand, scoping reviews are usually carried out to measure the degree, range and characteristics of study activity in a specific field, without assessing the quality of the studies. However, there are many characteristics of scoping reviews that separate them from traditional systematic reviews. In systematic reviews, quality filters are usually applied, but in scoping reviews, the quality of the published literature is not an initial determinant of inclusion. In addition to this, systematic reviews try to answer a clearly defined question and often use explicit methodologies to assess the quality of included articles. In contrast, scoping reviews are generally conducted to examine the extent, range and nature of research activity in a particular field without necessarily delving into the literature in-depth [17,18]. In case of SMA the information is sought from a potentially large and diverse body of literature concerning the nature of the topic which is broad. This necessitates to do scoping review whereas in systematic review it is intended to assemble pragmatic evidences from a relatively small number of studies related to a specifically focused research question

## 2. Methodology

In this scoping review study, a step-by-step methodological framework developed by Arksey and O’Malley was used [16] The methodology was strengthened by implementing the recommendations made by Levac et al. (2010) [19]. This methodology was also applied in a number of other studies [20,21,22]. The steps involved in the scoping review were determining the study query; identifying relevant research; research selection; charting; collecting; summarizing the data; and reporting the results.

### 2.1. Identifying the Research Question

According to the recommendations of the Arksey and O’Malley framework, all aspects of the research area should be considered to develop a question that generates a breadth of coverage. After analyzing the literature, we formed our overriding study question as follows: “What is the evidence of the main reasons, determinants and patterns for SMA by the lay public from low- and middle-income countries?”

We identified five areas of interest:

- What is the prevalence of SMA?
- What are the common causes of self-medication with antibiotics?
- What are the common patterns associated with self-medication with antibiotics?
- What are the sociodemographic factors associated with self-medication with antibiotics?
- What are the predictors for self-medication with antibiotics?

### 2.2. Inclusion and Exclusion Criteria

Inclusion criteria were specified as follows: papers written and available in English describing the main research study, using either a quantitative or qualitative study method or a combination of both. The exclusion criteria were developed during the title and abstract examination phase and applied at the full review stage, with one more additional criterion being included. The studies could not be performed on participants younger than 18 years old, students, inside universities, colleges, schools or institutes or on individuals with a medical background (doctors, pharmacists, nurses, para-medical staff). In other words, studies particularly performed on SMA among the general adult population were included in this scoping review study, and the search was also narrowed down to the articles published in the last 12 years. Finally, studies performed in low- and middle-income countries were included. To determine which countries should be included, we used the World Bank and International Monitoring Fund (IMF) list of low- and middle-income countries.

### 2.3. Relevant Literature Identification

For the scoping review to be in-depth, the Arksey and O’Malley recommended searching a number of literature resources, which included electronic directories and reference list of relevant literature [16]. We approached this scoping review study by including a number of different phases, first focusing on electronic literature databases including Google Scholar, ScienceDirect, ProQuest and PubMed. The keywords used for the search using Boolean operators were “Self-medication”, “antibiotics”, “sociodemographic factors”, “rational use”, “antibiotic misuse”, “patterns”, “reasons” and predictors. The online search was carried out from August 2018 to October 2018. A final online search was conducted in 2019 to find any potential new studies published.

### 2.4. Screening and Selection of Relevant Literature

The articles were reviewed by reading the titles as well as their abstracts. A specific screening procedure was utilized to evaluate the relevance of the selected literature. Studies were eligible for inclusion in this scoping review if they were conducted according to the scoping review methodology. To identify relevant studies, we examined every single study acknowledged for the complete review. All studies were examined in batches and upon conclusion of every fresh batch, all research team members met to examine decisions about the inclusion or exclusion of studies. If there was any disagreement between research team members at the title and abstract assessment phase, it was solved through consensus.

### 2.5. Charting, Collating and Summarizing the Results

Data were collated, summarized quantitatively and analyzed thematically to identify recurrent patterns in the selected articles. In the first step, relevant data were extracted from sources. To chart this data, a spreadsheet was made and utilized by each and every member of the research team. Extracted data included the study design, the objectives of studies, conclusions, outcomes and any information relevant to answering the scoping review questions. Second, data were charted based on the key themes identified including the prevalence of SMA, common causes of SMA, common patterns associated with SMA, sociodemographic factors associated with SMA and predictors of SMA. Finally, all research team members worked together to figure out the necessary strategies for potential future research by determining knowledge gaps. The results obtained from the included studies were summarized descriptively.

## 3. Results

**Figure 1:**
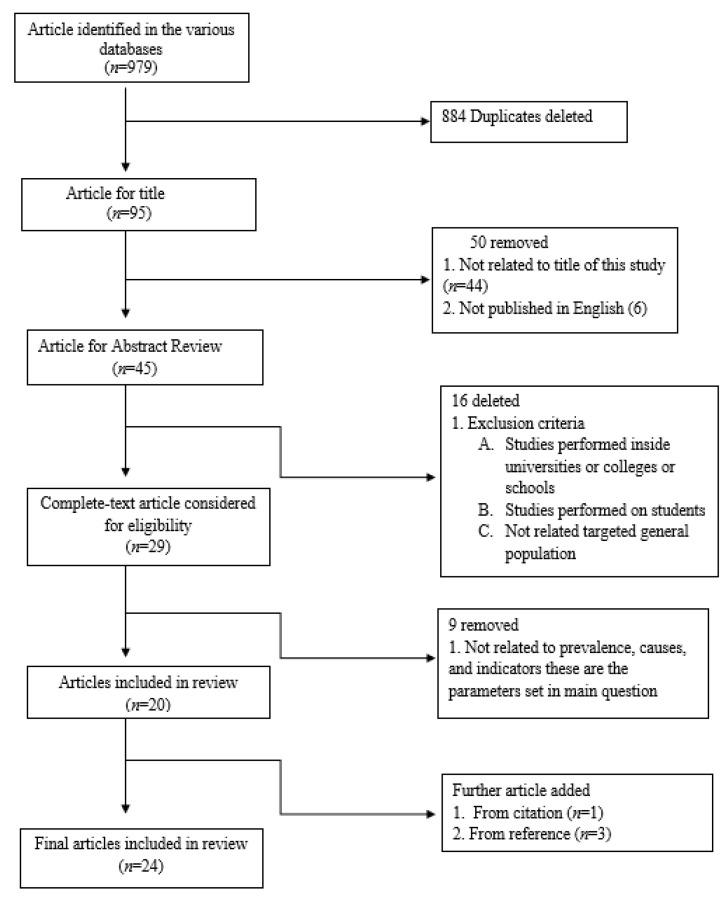
Flow diagram of study selection.

Originally, 979 content articles were identified and 95 remained after eliminating duplicates. These 95 articles were further reviewed by title which excluded 45, leaving a total of 45 articles for abstract review. The abstract screening method further excluded an additional 16 articles, leading to 29 full-text articles that were assessed for eligibility. Finally, 20 complete articles fulfilled the stipulated requirements for inclusion. After analyzing the reference lists of the included articles, four extra eligible articles were found, producing a total of 24 content articles. Figure 1 shows this article selection process.

### 3.1. Characteristics of Included Studies

A total of 24 studies were identified and included in this scoping review study; the characteristics of the included studies are presented in Table 1. These studies were executed using different methodologies such as cross-sectional [12,13,14,23,24,25,26,27,28,29,30,31,32,33,34,35,36,37,38,39], quantitative, [40,41,42] and mixed-methods or observational designs [23]. The number of participants in these studies ranged from 100 to 2696 and publication dates ranged from 2009 to 2018. Data on attitude, prevalence, sociodemographic factors, predictors and causes of SMA were collected from self-completed questionnaires [13,14,23,24,25,26,27,29,30,31,33,37], face-to-face interviews [32,36,38,40,41,43] and observer-rated compliance with recommendations [23]. Four of the studies were undertaken in Saudi Arabia [28,29,30,39], three in Pakistan [12,13,42], two studies in Jordan [40,41] and Turkey [38,41] and one each from Bangladesh, [24] Bulgaria [23], Beirut, [32] Cameroon [33] Columbia, [34] Greece, [25] Guatemala, [35] Haiti, [36] Indonesia [26] Cyprus, Egypt, Lebanon, Libya, Tunisia, [41] Nigeria, [14] Poland, [37] Portugal, [27] Romania, [43] and the United Arab Emirates [31]. All studies were conducted on the lay public from different countries and focused on the non-prescription use of antibiotics.

### 3.2. Prevalence of Self-Medication with Antibiotics

The prevalence of SMA was found to be low in middle-income countries, with wide differences among the affected countries—only 7.3% in Indonesia [26] and 26.9% in Bangladesh [24], to as high as 81.23% in Pakistan [13]. Six studies were identified from the Middle East and the overall frequency rate of SMA in Middle Eastern countries was highest in Saudi Arabia with 80.6% [30] and lowest in Jordan with 40.7% [25]. The prevalence of SMA in Europe was shown to be greater in Southern European countries, including Greece with 76.2% and Portugal with 18.9% [25,27] accompanied by SMA rates in Eastern European countries, specifically in Romania and Poland, with 41.1% Studies performed in central African countries showed a high prevalence rate in Nigeria with 82.2% and a low SMA rate in Guatemala with 79% [14,35].

### 3.3. The Distribution Pattern of the Most Commonly Used Antibiotics for SMA

In this review study, it was shown that in Indonesia, Pakistan and Bangladesh, the most frequently purchased antibiotic overall was amoxicillin (77%, 52%, 10.3%), followed by ampicillin, fradiomisin–gramisidin, tetracycline, ciprofloxacin, co-trimoxazole, cefadroxil, cefixime and azithromycin. On the other hand, antibiotics that were bought in the lowest amounts were flucloxacillin and cefuroxime [13,24,26]. In this review, six studies were conducted in the Middle East, and amoxicillin was the found to be the most commonly used antibiotic for SMA [29,30,31,40]. Among the Southern and Eastern European countries, broad-spectrum penicillins were utilized more significantly for SMA than in Northern and Western European countries [25,27,37,43]. While the most commonly used antibiotics for SMA among the people of Cameroon, Guatemala, Nigeria and Egypt were sulfamethoxazole/trimethoprim, metronidazole, cotrimoxazole and amoxicillin [14,33,35,41].

### 3.4. Factors Associated with SMA

The commonly reported factors associated with SMA were as follows: past effective use, level of education, female gender, male gender, age group and middle (income) class and job type. Studies done in Africa reported a low degree of education, severity of disease (mild to severe), female gender, age group (≥45 years) and middle class as the most common determents for SMA. Similarly, in Middle Eastern countries, level of education, age (18–39 years) and middle income were common factors associated with SMA.

### 3.5. Common Indications Related to SMA

Antibiotics were found to be primarily self-administered for bacterial and non-bacterial infection. Antibiotics used for non-bacterial infections were found to be ringworm, headache, allergy, rash, fatigue and asthma. Antibiotics used by people for bacterial infections were pharyngitis, dysentery, food poisoning, sinusitis, tonsillitis, eye infection, prostatitis, itching, acne, discomfort when urinating, influenza, toothache, gastrointestinal infections, upper respiratory tract infection, ear infection, dysuria, dermatitis, skin disease, urinary tract infection, diarrhea, fever, vaginal discharge, rheumatism, burning micturition, common cold, cough and sore throat and skin sepsis.

### 3.6. Information on and Source of Antibiotics for Self-Medication

Details related to antibacterial agents may be obtained from different sources in different countries. Most of the studies included in this review reported that drug retailers or pharmacists and family members, relatives or close friends are the key sources of information about SMA. The additional reported resources included previous successful use of antibiotics and medication leaflets. The antimicrobial medications used in SM were found to be acquired from numerous different sources, e.g., pharmacies, leftover drugs, private hospitals and gifts from close friends. In this scoping review, we found the following common sources: pharmacies, leftover medicines from the drug store, chemist shops, corner stores, street vendors, marketing agents, previous doctor’s prescription and relatives and friends. Most individuals reported that SMA is important because it is time saving, provides quick relief from illness, removes financial constraints, because they lack a trusted medical doctor and because it is convenient.

## 4. Discussion

### Main Findings

The main finding of this scoping review is that there are many published studies indicating that the prevalence of SMA is alarmingly high among the lay population in LMICs. The lay public often does not have adequate knowledge about antimicrobial agents and also has a low understanding about (infectious) disease processes. However, the lay public commonly obtains antimicrobial agents due to following the advice of other people and use them as over-the-counter (OTC) medicines. Results of the studies included in this review also indicate that individuals with a high level of education are less likely to self-medicate antibiotics. So, the implementation of educational policies could increase health literacy among communities and significantly minimize SMA. SMA could also be reduced by providing easy and quick access for individuals to primary healthcare centers [44]. However, although most LMICs do not have sufficient resources to provide easy access for individuals to primacy healthcare, most of the population has easy access to antibiotics as OTC medicines, and some individuals also utilize prescription-only antibiotics for longer periods of time or without any medical supervision. Such bad practices do not benefit patients—especially in the case of antibiotics—as they are associated with dangerous effects at both at the individual level (adverse events) and community level (the development of antibiotic resistance). If people utilize antibiotics in an appropriate manner, drug resistance as well as the death rate will reduce. A systematic analysis on the global burden of disease from lower respiratory infections showed that if people have access to appropriate antibiotics, the death rate can be reduced by up to 30.2% [45]. However, a recently published review showed that LMICs have insufficient resources to cope with respiratory diseases [46] and this is one of the common determinants for SMA among the lay public of LMICs. The decision of patients to use prescription-only antibiotics without any proper advice from healthcare professional is a reflection of their country’s healthcare system quality and economic status [47]. Consequently, considering all these factors playing vital roles, the design of new interventional programs to reduce irresponsible behaviors towards SMA is important. The current scoping review study also shows an approximation of the prevalence of SMA and its related results inside communities. All included studies showed a prevalence for SMA of 38.82% overall. This is almost consistent with the findings of the first review on global antimicrobial SM, which showed an SMA prevalence of 39%. This prevalence was estimated from countries where antibiotics are labeled as prescription-only medications and people are undergoing SMA without proper medical prescriptions [48]. This scoping review also revealed that the prevalence of SMA is higher in the South Asian and Middle East countries because of financial constraints of the inhabitants, who cannot afford a complete course of antibiotics. Another factor related to SMA is the level of education. A low level of education possibly increases the chance of risks connected with the use of non-prescription antibiotics. For instance, development of the antimicrobial resistance (AMR) is a consequence of irrelevant medicine utilization that is typically connected with SMA and which causes a greater mortality rate in LMICs when compared with developed countries [49]. This scoping review study also reports that the prevalence of SMA varies around the world. This may be because of the major difference in the capability to enforce regulations on SMA in various developing countries. Even so, there is substantial heterogeneity inside the results of studies included in this scoping review (e.g., the prevalence, pattern and factors related to SMA); therefore, we were not able to incorporate the included studies in a meta-analysis.

The studies included in this review also reported the most common reasons for SMA. Most of the participants report that they undergo SMA because it is convenient, affordable and time-saving. These positive aspects further encourage community members to undergo SMA and motivate them to take care of self-diagnosed health problems. Another common reason for SMA is the easy availability of antibiotics without prescription, an underlying challenge to health care systems that is prevalent among LMICs. This may be due to the lack of potentially strict policies inside the healthcare systems [50]. In addition to this, there is an irregular supply of medications to the public healthcare centers in LMICs, which hinders the community from accessing public healthcare facilities. Together with these common reasons, most LMICs also have high rates of infectious diseases which makes the private sector a vital substitute for the community to gain access to primary healthcare facilities [51]. Yet, a number of studies pointed out that the possible benefits linked with SMA can only be achieved by utilizing antibiotics safely, effectively and responsibly [13,23,24,28]. The key determinants of SMA in LMICs are level of education, the intensity of illness, financial status and previous successful use of antibiotics. Many individuals usually start treating themselves without consulting with a healthcare professional because of consultation costs and travel expenses [52]. By summarizing the finding, in this scoping review study we identified that the key determinants from the included studies are prior successful use of antibiotics and consensus among individuals in the community that they can manage any succeeding illness or disease without consulting a physician. This is certainly a potential risk factor for improper medication utilization, as most consumers lack understanding of the disease procedure and also have a low understanding of medications used to manage their self-diagnosed disease. Furthermore, people are likely to use antibiotics without proper guidelines. For example, they may stop taking antibiotics once symptoms start improving [13], which results in their delaying relevant treatment and possibly allowing the disease to become more severe. Due to this delay in treatment, a high mortality rate was noted among individuals suffering from treatable diseases [53]. This scoping review study also indicates that improper practices, e.g., completing full course of antibiotics, sharing medicines and stopping medication when symptoms start to improve, are carried out in certain communities [13,24,30]. The various studies included in this scoping review study reported that most individuals utilize more than one antibiotic agent for SM, and this practice sometimes makes the condition worse (due to the additive effect of the adverse events), rather than beneficial. All of these factors together can result in maltreatment, drug interactions, adverse drug reactions and possible increases in antibiotic resistance [51,54,55,56]. In this scoping review study, we also identified that information regarding SMA is obtained from a variety of different sources, such as drug retailers, recent antibiotic prescriptions, earlier successful use, relatives or friends and drug leaflets. Lastly, drug leaflets could be an important method through which individuals could obtain information, but their poor legibility makes them challenging to use [6]. Additionally, the high rate of illiteracy in LMICs [51] limits the usefulness of leaflets as a source of information. Providing information leaflets in the native (indigenous) languages of the population and using accessible language without complicated medical terminology could enhance the usefulness of these drug leaflets [51].

## 5. Conclusions

Evidence obtained from the included studies shows that SMA is highly prevalent among individuals living in low- and middle-income countries and is often connected with improper use of these medicines, which is a leading cause of resistance. This review mainly focused on SMA practices among the lay public, and results from included studies showed that most of the people studies use antibiotics because they are time saving and cheap. In addition—based on findings of the included studies—the most significant “medical” reasons for SMA in low- and middle-income countries are fever, upper respiratory tract infection, common cold and sore throat. Most of the people studied obtained information about antibiotics from pharmacists or family member. Therefore, pharmacists should be encouraged to educate patients about proper use of antibiotics. Furthermore, this scoping review found that male gender and lower educational level have significant associations with SMA. Thus, interventions should focus on the level of education and gender as key areas. Lastly, the detrimental effects of SMA on health, social and economic perspectives warrant appropriate planning and policy-making. Furthermore, by improving public access to primary healthcare facilities, implementing educational interventions, enforcing the strict regulations on non-prescription antibiotic use. Reducing the burden of infectious diseases may help to mitigate the challenge of SMA.

## 6. Future Recommendation

This review provides an overview of SM antibiotic utilization practices and could, therefore, be used to design interventions. This scoping review found variability among the included studies in terms of designs and outcomes. Therefore, there is a need for multinational studies based on standardized methodological approaches to get a better comparative understanding of the global practices and prevalence of SMA. To ensure appropriate utilization of antibiotics, multifaceted interventions that can target the healthcare system, lay public and healthcare professionals—including pharmacists—are needed. In addition to this, future research is needed to gain more insight into the influences of factors and determinants associated with SMA at the healthcare system level and healthcare professionals in order to design effective interventions in these settings. Furthermore, gearing the power of artificial intelligence to combat the menace of self-medication with antibiotics will be a welcome move.

## 7. Strength and Limitation

To best of our knowledge, this is the first scoping review on the evidence of main reasons, determents and patterns for SMA by population among developing countries. We were able to include a number of studies examining SMA practices at lay public level. However, studies performed on students and on persons having medical background were excluded from this review. Despite our comprehensive search of the database, it is possible that we did not find all related information regarding SMA because we restricted our research to Google Scholar, ScienceDirect, ProQuest and PubMed only. At the same time, we found a wide variety of studies that focused on SMA. Another limitation is that we searched only for publications that were written in only the English language. A third limitation is the potential lack of comparability between included studies due to different outcome measures they presented and the variety of study designs.

## Figures and Tables

**Table 1:** Characteristics of included studies.

Author (Year), Country	Aim	Sample Size	Study Design, Data Collection, Instrument and Setting	Conclusion	Results
Dimova et al. (2014) Bulgaria [23]	To examine the attitudes and SM patterns that associated with the utilization of antibiotics between the Bulgarian general population and also their determinants.	Patients (*n* = 1044) Male (*n* = 482) Female (*n* = 562)	Cross-sectional and observational study design; survey performed through mail (closed and open-ended questions)	High prevalence of SMA due to sociodemographic factors.	The observed SMA rate was 43%. The younger, female, students and employees tended to have a much higher SM rate. The most consistent patterns that associated with the SM practice were fever (22.0%), discomfort when urinating (8.2%) and sore throat and cough (12.7%).
Biswas et al. (2014) Bangladesh [24]	The aims and objective of the current study was to explore the prevalence of SMA for treating various illnesses by the peoples	Patients: (*n* = 1300)	Cross-sectional survey (self-completed questionnaire) public places	SMA is a common problem among the Bangladesh population. Authority must take strict action to control the distribution and sale of antibiotics to decrease the incidence of misuse.	347 (26.6%) experienced SMA and the highest percentage of SMA was (50.4%) metronidazole. The key reasons for the SMA were past experience (45.8%) and recommendations from others (28.2%). While the primary reasons behind SMA were food poisoning and diarrhea (36.0%), toothache (9.2%), fever, cough and cold (28.2%), infection (12.9%), acne (4.3%), irritable bowel syndrome (3.4%), throat and ear pain (2.3%).
Cheaito et al. (2014) Beirut [32]	This study was performed to evaluate SMA in the overall population and its correlated factors.	Participants: (*n* = 319) Male (*n* = 143) Female (*n* = 176)	Cross-sectional: (face-to-face interview using standardized questionnaire) Pharmacies	SMA was a frequent issue in the Beirut area. Authorities must endorse interventions to minimize antibiotic misuse.	The prevalence of SMA was 42% and the pharmacists were the primary source of buying antibiotics (18.8%). SMA was most commonly associated with sore throat symptoms and the most frequently used antibiotic was amoxicillin.
Ngu et al. (2018) Cameroon [33]	The objectives of this study were to evaluate the incidence of SMA and recognize the factors that contribute to SM among adult patients with respiratory tract illness in Cameroon.	Participants (*n* = 308) Male (*n* = 138) Female (170)	Cross-sectional: (self-completed questionnaire) Hospital	Outcomes revealed that SMA for respiratory tract infections (RTIs) is a widespread practice that needs to be addressed properly.	The prevalence of SMA among individuals with RTIs was 41.9%. Individuals with a history of pulmonary tuberculosis (TB) were considerably less likely do SMA. The most consistent way to obtain antibiotic for SM was pharmacies (62%). Cotrimoxazole (38.8%) and Amoxicillin (26.4%) were the most frequently used antibiotics.
Landers et al. (2010) Columbia [34]	The objective of this study was to find out the level of antibiotics utilization meant for upper respiratory tract infections.	Participants (*n* = 100)	Cross-sectional: self-completed questionnaire (door-to-door survey)	Researchers suggested that there is a need to address social and cultural contexts to improve antibiotic usage among the general public.	Overall, participants 34% using antibiotics for upper respiratory tract infection (URT). Sevety-three percent of women in this study reported that at least one member of the household used antibiotics during the past season. Among adult individuals, SMA was reported 67.2%, however only 2.4% among children.
Skliros et al. (2010) Greece [25]	To evaluate prevalence Self-SMA without prescription.	Participants (*n* = 1139) Male (*n* = 545) Female (*n* = 595)	Cross-sectional: self-completed questionnaires. Carried out in 6 rural health centers in southern Greece.	According to researchers a higher percentage of the rural adult population was consuming antibiotics without any proper prescription.	Total (76.2%) participants self-medicated, while 508 (66.6%) buy medicine from pharmacies. The most commonly used antibiotic was amoxicillin (18.3%), then cefaclor (9.7%), after that cefuroxime (7.9%), finally cefprozil (4.7%) and ciprofloxacin (2.3%). While most frequent reasons for SMA were highest for fever (41.2%), common cold (32.0%) and lowest for sore throat (20.6%).
Ramay et al. (2015) Guatemala [35]	To explore and compare the magnitude of SMA and the characteristics among socioeconomic communities	Participants (*n* = 418) Male (*n* = 126) Female (*n* = 292)	Cross-sectional: (descriptive study) Pharmacies	Researchers recommended that there is a need for interventional programs for the sale of antibiotics and also for interventional programs for pharmacists to play effective roles in antibiotic use.	The higher percentage of SMA was reported in the suburban area 79% and while 77% in the city area. In both places, amoxicillin was the most commonly bought antibiotic for SM. Flu-like symptoms were the most common in the Suburban (33%) and, while in metropolitan areas pain and fever (32%) were the most common indication for SMA.
Moise et al. (2017) Haiti [36]	To evaluate the prevalence of SMA, Factor associated with it and knowledge to examine SMA in Haiti.	Participants (*n* = 200) Male (*n* = 76) Female (*n* = 124)	Cross-sectional: (face-to-face interview using standardized open-ended and close-ended questionnaire) Hospital	SMA is a common practice among the Haitian population and it is essential to improve public awareness about the dangers practice of SMA and enforce the current law to minimize the consumption of over-the-counter antibiotics.	Among the study sample, 45.5% practiced SMA and It was less prevalent among individuals having good educational background (23.1%). While key reasons for SMA were vaginal itching and mild infection symptoms (44.4%, 28.6%). amoxicillin was reported highest used antibiotic at 67.0%.
Widayati et al. (2011) Indonesia [26]	The aim of study was to evaluate SMA prevalence rates and patterns associated with it.	participants (*n* = 559) Male (*n* = 219) Female (*n* = 259)	Cross-sectional: population-based survey (closed-ended self-completed questionnaire). Public places	Researchers identified that the key reason behind SMA was the past experience and a higher percentage of SMA was noted among males.	The prevalence rate among the population was 7.3% and around (6%) doing SMA. while 7% of participants was used prescription-only antibiotics; Amoxicillin (77%) was the commonly used antibiotic and 64% bought medicine from pharmacies. Headache, fever, common cold, toothache, cough, itching, sore throat was the most common indication for antibiotics use.
Sawair et al. (2009) Jordan [40]	To determine the extent of SMA among the Jordanian general public.	Patients (*n* = 477) Male (*n* = 220) Female (*n* = 257)	Quantitative (face-to-face interview using a standardized questionnaire) Dental clinics	SMA is apparently common among Jordanians and there is a need to design antibiotic educational campaigns to educate the peoples about their unwanted effects.	The rate of SMA among the general public was 40.7% and peoples with low income and aged between 36–55 years mostly do SMA. Antibiotics were mainly utilized for dental infection, common colds, sore throats and pharmacies were the primary sources (53.6% cases). While Amoxicillin (37.6%) was a commonly used antibiotic.
Scicluna et al. (2009) Euro-Mediterranean Countries [41]	The aim of study was to evaluate and identify SMA rates inside Mediterranean countries.	Participants (*n* = 1705)	Quantitative (short structured interviews using questionnaire) Hospitals and private clinics	Researchers found that in the southern and eastern region of Mediterranean countries non-prescribed antibiotic consumption was much higher inside ambulatory care.	In Cyprus, the SM rate was 19.1%, while in Lebanon it was 37% and in Jordan 70.7%. URTs symptoms was the most consistent known reasons for SMA. A total of 48.4% of the peoples responded that they have leftover antibiotics in their homes.
Abdulraheem et al. 2016 Nigeria [14]	To estimate the prevalence of SMA in a sample of the rural population and to evaluate sociodemographic aspects linked to this practice.	Participants (*n* = 1150) Adults (*n* = 602) Adults Parents (*n* = 548)	Cross-sectional: (self-completed questionnaire) Primary health care center	SMA is a critical problem in Nigeria and needs substantial attention.	The prevalence of SMA was 82.2% and the antibiotics most regularly used for SMA were ampicillin (20.3%), second, sulfamethoxazole/ trimethoprim mixture (14.2%), third, metronidazole (13.9%) and tetracycline (13.1%). A cough with mucus (30.1%), sore throat (23.7%), fever (20.7%), dysuria (10.6%) skin sepsis (7.5%) and vaginal discharge (7.4%) were the most typical reasons for SMA.
Nazir et al. (2016) Pakistan [42]	To assess various factors that lead to SMA, which may cause AMR and cause a hindering effect in healthcare.	Participants (*n* = 527)	Cross-sectional: (standardized questionnaire) Public places	A need for public health policy through drug regulatory authorities, public education campaigns about AMR and appropriate utilization of antibiotics were highlighted.	SMA was reported by 26% participants, with an increased prevalence in males compared to females (48% vs 38%, respectively). The primary reason for SMA was the previous encounter with the same antibiotic (68%). The mostly used antibiotics was amoxicillin-clavulanate (40%) and the main indications for self-medicine were sore throat (29%) and flu (24%). Out Of the 527 respondents, just 20% were alert to AMR.
Khan et al. (2011) Pakistan [12]	To evaluate the SMA rate among the study was the urban general public and to identify the factors associated with this practice.	Participants: (*n* = 744) Male (*n* = 350) Female (*n* = 394)	Quantitative Descriptive study Private clinics	Researchers concluded that healthcare facilities in Peshawar are good and the general public also has easy access to them, despite that people’s utilization of non-prescription antibiotics.	The prevalence towards SMA was (69%) and the antibiotics most regularly used at the first place were amoxicillin/clavulanic acid (45%), then ciprofloxacin (31%), after that sulfamethoxazole/trimethoprim (18%) and finally place clarithromycin (5%). The main indications for SMA were fever (70%), then sore throat (22%) and common cold (8%).
Bilal et al. (2016) Pakistan [13]	The study was aimed to explore the prevalence rate and practice towards SMA between general public livening in rural regions of Sindh province.	Participants (*n* = 400) Male (*n* = 263) Female (*n* = 137)	Cross-sectional: (Self-completed; close-end questionnaire) Hospital	The SMA rate was higher in rural regions of Sindh. The government must enforce stricter laws to control this practice. Lastly, by providing cost-effective treatment to the public sector, SMA can be considerably decreased.	325 rural dwellers reported usage SMA during the past 6 months period with a prevalence rate of 81.25%. The mostly bought antibiotics were amoxicillin (52.0%) accompanied by tetracycline (16.9%), then ciprofloxacin (14.8), after that co-trimoxazole (11.4%) and ampicillin (8.3%) finally place. Common indications for SMA was flu (60.0%) and (88.0%) also reported that economic reasons for SMA.
Muras et al. (2013) Poland [37]	The purpose of this study was to find out the prevalence rate for SMA intended for RTIs and also identified factors associated with this practice.	Participants (*n* = 891) Male (*n* = 304) Female (*n* = 587)	Cross-sectional: (self-completed questionnaire) Family medicine Clinics	SMA for RTI was frequent among the general population and according to researchers, this maybe because of the people’s opinion that the drugs treat almost all infections.	Overall, 41.4% of participants reported SMA for respiratory tract infection (RTI) and the second most common indication reported for SMA was influenza or flu-like disease (43.9%). The primary sources for antibiotics was drugs kept at home obtained from previous prescriptions (73.7%). While (13.5%) bought antibiotics from the pharmacy or got it from friends or their family members (12.7%).
Ramalhinho et al. (2014) Portugal [27]	To evaluate the prevalence of SMA and measure the predictive factors connected with such SM.	Participants (*n* = 1192) Male (581) Female (611)	Cross-sectional: a population-based survey. (self-completed questionnaire) Public places	SMA was greater among older males than young adults. Researchers also reported that gender and age were key determinants for SMA.	218 respondents (18.9%) admitted that they previously took antibiotics without a prescription and 267 (23%) participants noted that they were keeping leftover antibiotics in their homes. The factors that affect SMA rate were age; in particular 18–34 years and in the 50–64; while these practices less seen in 44–49 age groups.
Bianca et al. (2013) Romania [43]	The purpose of this study was to explore factors that responsible for OTC purchasing of antibiotics and SMA in Romania.	Participants: (*n* = 20) In-depth interview (*n* = 10)	In-depth qualitative face-to-face interview; (snowball sampling technique)	This study highlighted that population generally dissatisfied with their medical situations and the healthcare program.	The participants that self-medicated mentioned kidney and urinary infections as the main indication. Antibiotics used for these diseases were amoxicillin, cephalexin and norfloxacin. Flu and cold, treated by over-the-counter medicines and antibiotics used were amoxicillin, cephalexin and ampicillin.
Yousif et al. (2015) Saudi Arabia [30]	Exploring the prevalence of SMA and also determine its determinants.	Participants (*n* = 400) Male (*n* = 228) Female (*n* = 291)	Cross–sectional: Population-based survey. (Close-ended self-completed questionnaire) Public places	SMA was prevalent and there is a need for general public education campaigns along with stringent strategies to decrease or restrict OTC sales of antibiotics inside community pharmacies.	Almost 315 (80.6%) individuals accepted that they are doing SMA and 235 (74.6%) participants practiced it due to easy availability. Around 123 (39.0%) Participants doing SMA and they thought they treat infectious successfully with the help of antibiotics. while 146 (46.3%) individuals were not sure about this and 46 (14.6%) responded: “they cannot”.
Alghadeer et al. (2018) Saudi Arabia [29]	This research aimed to find out the prevalence rate and common indication for SMA.	Respondents (*n* = 1264) Male (*n* = 345) Female (*n* = 919)	Cross-sectional: An online survey using the snowball technique. (self-completed questionnaire)	The high prevalence rate for SMA demands to take significant actions by authorities to implement strict laws to prevent the sale of antibiotics without a proper prescription.	Overall, 34% of participants consumed antibiotics without a prescription and around 81.3% of individuals recognized that it could be unsafe. The common antibiotics utilized were amoxicillin/clavulanic acid (45.1%) in the first place, amoxicillin alone (39.9%), then azithromycin (16.8%), after that cefuroxime (9.7%) and cephalexin (5.7%) finally place. The most typical reasons for antibiotic were tonsillitis (76.7%). The main source of SMA were drugs obtained earlier through a doctor’s prescription (36.6%).
Al Rasheed et al. (2016) Saudi Arabia [28]	To determine the common predictors and prevalence towards SMA.	Patients: (*n* = 681) Male (*n* = 523) Female (158)	Cross-sectional: (closed and open–ended questionnaire) Hospital	High prevalence rate towards SMA was noted and the researchers suggested that education campaigns are required for appropriate antibiotic utilization.	The prevalence rate for SMA was 78.7% and amoxicillin was the most utilized antibiotic with the overall prevalence rate of (22.3%). Advice from friends on SMA and pharmacy proximity to individuals were the most common predictors for SMA.
Al-Qahtani et al. (2018) Saudi Arabia [39]	To determine the prevalence of SMA and factors related to this behavior	Participant (*n* = 519) Male (*n* = 255) Female (*n* = 294)	Cross-sectional: (Self-administered questionnaire) Hospital	A high prevalence rate toward SMA was noted among the lay population. Researchers suggested that by using media awareness campaigns, authorities can decrease SMA and the adverse events related to it.	SMA prevalence rate was 40.8% and elder people were mostly associated with SMA. While the most common disease was RTIs (73.2%) and most of the peoples obtained antibiotics from pharmacies (82.8%). Past good experience with SMA was the main reason for continuous SM (67.2%)
Ilhan et al. (2009) Turkey [38]	The purpose to find out the frequency and know the reasons for SMA.	Participants (*n* = 2696)	Face-to-face interview (using standardized questionnaire) Primary health care center	Researchers indicated the necessity of strict laws to regulate antibiotic sales and also identified the need for education policies in the community to reduce inappropriate use of antibiotics.	The most common reasons for SMA were sore throat (59.6%), then fever (46.2%) and finally cough (40.0%). Other common indications were rheumatism, exhaustion and dental infection. According to age ranges, the most prevalent age group was 40–49 with 23% doing SMA and the least rate noted around 11.8% among 60–69 years old.
Abasaeed et al. (2009) United Arab Emirates [31]	The main purpose was to evaluate the prevalence of SMA.	Participants: (*n* = 860) Male (*n* = 566) Female (294)	Cross-sectional: population-based survey (self-completed questionnaire) Public places	The outcomes indicated that SMA is a consistent issue in the general public of Abu Dhabi. Interventions are required to cut-down the improper use of antibiotics.	In both genders overall, 485 (56%) admitted that they consumed antibiotics during the past year. Education level has a significant impact on SMA, and amoxicillin was the antibiotic most commonly used (46.3%). The common reason for SMA were influenza, toothache, ear, upper respiratory and gastrointestinal infection.
